# Alcohol Triggers the Accumulation of Oxidatively Damaged Proteins in Neuronal Cells and Tissues

**DOI:** 10.3390/antiox13050580

**Published:** 2024-05-08

**Authors:** Anusha W. Mudyanselage, Buddhika C. Wijamunige, Artur Kocoń, Ricky Turner, Denise McLean, Benito Morentin, Luis F. Callado, Wayne G. Carter

**Affiliations:** 1 Clinical Toxicology Research Group, School of Medicine, University of Nottingham, Royal Derby Hospital Centre, Uttoxeter Road, Derby DE22 3DT, UK; wijesekara@agri.sab.ac.lk (A.W.M.); buddhikawijamunige@agri.sab.ac.lk (B.C.W.); artek.1993@googlemail.com (A.K.); ricky.turner@nhs.net (R.T.); 2 Department of Export Agriculture, Faculty of Agricultural Sciences, Sabaragamuwa University of Sri Lanka, Belihuloya 70140, Sri Lanka; 3 School of Life Sciences, University of Nottingham, Nottingham NG7 2UH, UK; denise.mclean@nottingham.ac.uk; 4 Section of Forensic Pathology, Basque Institute of Legal Medicine, E-48001 Bilbao, Spain; morentin.b@justizia.eus; 5 Department of Pharmacology, University of the Basque Country-UPV/EHU, E-48940 Leioa, Spain; lf.callado@ehu.eus; 6 Centro de Investigación Biomédica en Red de Salud Mental (CIBERSAM), Spain

**Keywords:** alcohol, alcohol-related brain damage, developmental neurotoxicity, oxidative stress, protein carbonylation, reactive oxygen species

## Abstract

Alcohol is toxic to neurons and can trigger alcohol-related brain damage, neuronal loss, and cognitive decline. Neuronal cells may be vulnerable to alcohol toxicity and damage from oxidative stress after differentiation. To consider this further, the toxicity of alcohol to undifferentiated SH-SY5Y cells was compared with that of cells that had been acutely differentiated. Cells were exposed to alcohol over a concentration range of 0–200 mM for up to 24 h and alcohol effects on cell viability were evaluated via MTT and LDH assays. Effects on mitochondrial morphology were examined via transmission electron microscopy, and mitochondrial functionality was examined using measurements of ATP and the production of reactive oxygen species (ROS). Alcohol reduced cell viability and depleted ATP levels in a concentration- and exposure duration-dependent manner, with undifferentiated cells more vulnerable to toxicity. Alcohol exposure resulted in neurite retraction, altered mitochondrial morphology, and increased the levels of ROS in proportion to alcohol concentration; these peaked after 3 and 6 h exposures and were significantly higher in differentiated cells. Protein carbonyl content (PCC) lagged behind ROS production and peaked after 12 and 24 h, increasing in proportion to alcohol concentration, with higher levels in differentiated cells. Carbonylated proteins were characterised by their denatured molecular weights and overlapped with those from adult post-mortem brain tissue, with levels of PCC higher in alcoholic subjects than matched controls. Hence, alcohol can potentially trigger cell and tissue damage from oxidative stress and the accumulation of oxidatively damaged proteins.

## 1. Introduction

Ethyl alcohol (ethanol) is the most widely imbibed, licit, psychoactive drug. Although drinking alcohol is an element of the social fabric of many cultures, there are serious health concerns and consequences that can arise from excessive alcohol intake [1,2,3]. The relationship between alcohol and human harm is complex and multidimensional but does increase monotonically with increased consumption [3]. The number of global deaths attributed to the harmful use of alcohol was over 3 million in 2016, constituting 1 in 20 deaths [4]. In terms of disability-adjusted life years (DALYs), over 5% of the global burden of disease is causally linked to alcohol usage [4,5].

The impact of alcohol on health relates to both the volume of alcohol consumed and the pattern of drinking, including the number of heavy drinking sessions [6]. Epidemiological analyses have established an association between alcohol usage and over 200 somatic diseases [7]. For some of these diseases, such as liver cirrhosis, a relative-risk dose response exists [8] but the relationship between alcohol intake and risk of disease is not uniformly dose-dependent in all tissues. For some tissues, a curvilinear relationship such as a J- or U-shaped curve may exist such that low to moderate drinkers have a reduced health risk compared with certain cohorts of abstainers. Although still a moot point, some epidemiological studies have suggested a protective benefit of low-level alcohol consumption for reduced risk of diabetes mellitus, ischemic heart disease, and dementia [7,9,10]. Nevertheless, the evidence base for long-term cognitive damage to alcoholics is considerable. Some epidemiological studies have suggested a reduced risk of development of dementia for certain minimal and light drinking cohorts when compared with abstainers, but many studies have concluded that heavy drinking is associated with an increased risk of dementia and cognitive decline [11,12,13,14,15,16,17].

In support of an association between excessive alcohol drinking and dementia, brain atrophy, damage, and neuronal loss have all been detected in many but not all post-mortem studies of brains of alcoholics [18,19,20,21,22,23,24,25]. Likewise, brain shrinkage of white and/or grey matter in response to longitudinal alcohol exposure has been detected using a range of in vivo imaging techniques [26,27,28,29,30,31,32,33,34]. Furthermore, specific localised volumetric reductions of subcortical structures including the prefrontal cortex and hippocampal regions have also been detected in alcoholics [30,32], and correlate with cognitive decline [32].

Adolescence is a period of notable vulnerability to the neurotoxic effects of alcohol, with binge drinking associated with reduced grey matter and detrimental effects on attention and cognition [35,36]. The elderly may also be more responsive to the toxic effects of alcohol [36], and there is a decline in brain structure with age that mirrors that observed in alcoholic patients [25]. Alcohol also has teratogenic effects, such that excessive maternal alcohol consumption during pregnancy impacts the neurodevelopment of the foetus and results in foetal alcohol spectrum disorders (FASD), and negative effects on cognition [36,37,38,39,40]. FASD is recognised by the presence of a range of impairments to growth, dysmorphia, and central nervous system (CNS) dysfunction, including deficits in cognition and neurobehavioural abnormalities as a consequence of brain damage [36,37,38,39,40]. Reduced grey and white matter contribute to the collective reduction in brain size for babies with FASD [39,40]. Alcohol may therefore be particularly neurotoxic during periods of neurodevelopment and in the elderly, and this could be mediated by mechanisms including cellular redox stress and induction of apoptosis [40,41,42,43].

Excessive alcohol exposure can result in a depletion of numbers of neurons (cell death), but alcohol also has a broad impact on neurocircuitry and plasticity [40,44] and these can diminish the functionality of surviving neurons [39,40,45]. Hence, to gain more insight into the effects of alcohol on newly differentiated neuronal cells, and the potential impact of oxidative stress, the toxicity of alcohol was directly compared between undifferentiated and differentiated SH-SY5Y cells. Neurotoxicity was assessed via quantitation of alcohol effects on cell viability, mitochondrial morphology and functionality, the induction of reactive oxygen species (ROS), and the accumulation of oxidatively damaged proteins. Studies were also undertaken to consider whether the oxidative damage observed in cells after alcohol exposure was mirrored by that present within human post-mortem brain tissue from alcoholics.

## 2. Materials and Methods

### 2.1. Cell Culture and Cell Image Capture

The SH-SY5Y human neuroblastoma cell line was purchased from the European Collection of Authenticated Cell Culture (ECACC) (ECACC-94030304). Experiments were conducted with cells from passages 13–14. SH-SY5Y cells were grown in the following culture medium: 43.5% Eagle’s Minimum Essential Medium (EMEM) (M4655, Sigma, Poole, UK) supplemented with 43.5% Ham’s F12 nut mix (217665-029, Gibco, Waltham, MA, USA), 10% heat-inactivated fetal bovine serum (FBS) (F9665, Sigma, Poole, UK), 1% MEM Non-Essential Amino Acid Solution (NEAA) (RNBF3937, Sigma, Poole, UK), 2 mM glutamine, and 1% penicillin–streptomycin solution containing 10,000 IU penicillium and 10 mg/mL streptomycin (p/s) (P4333, Sigma, Poole, UK) in 25 or 75 cm^2^ flasks (Thermo Fisher Scientific, Rochester, UK) at 37 °C with an atmosphere of 5% CO_2_ and 95% humidity, as previously described [46]. Cells were observed daily and grown until the cells reached approximately 80% confluence, after which the culture medium was refreshed every other day.

For differentiation, SH-SY5Y cells were seeded on either poly-D-lysine (PDL) hydrobromide (5 mg/mL) (P6407, Sigma, Poole, UK) coated 25 cm^2^ flasks (T25, 130189, Thermo Fisher Scientific, Rochester, UK) or in 96-well microtiter plates (6005649, Perkin Elmer, Groningen, The Netherlands) with 10% FBS media. After the cells had settled, they were grown to 60% confluency. The following day, the cells were treated with differentiation medium (10 µM all-trans retinoic acid (RA) (R2625, Sigma, Poole, UK) in low-serum SH-SY5Y medium (1% FBS) for 6 days and then treated with 20 ng/mL brain-derived neurotrophic factor (BDNF) (B3795, Sigma, Poole, UK) with low-serum medium containing RA for 2 more days, after which the cells displayed a fully differentiated morphology [46,47].

Cells treated with alcohol (10–200 mM) were monitored with an inverted microscope with phase-contrast optics (Olympus, DP70, London, UK) to compare the general morphological changes with untreated controls for both undifferentiated and differentiated cells at the end of the treatment period. Cells that were cultured in 12-well PDL-coated plates were used to study the neurite length changes in differentiated cells in response to 0–200 mM alcohol treatments. Cells were considered to be differentiated if each neuronal cell contained at least one component that was longer than its cell body [48]. The neurite lengths from 200 randomly chosen cells were measured in 5 selected quadrants per well using the neurite tracer tool from Image J (Image J 1.49k, National Institute of Health, Bethesda, MD, USA), in three independent wells for each treatment [49].

Untreated cells and those incubated with alcohol for 24 h were prepared for transmission electron microscopy (TEM) according to the methods described in [50]. In brief, after a 24 h incubation, the medium was removed and cells were washed with medium containing fixative (3% glutaraldehyde in 0.1 M cacodylate buffer). The media–fixative solution (1:1 (*v*/*v*)) was then replaced with fixative alone, before the cells were fixed in an incubator for 1 h at 37 °C. Cells were scraped into the fixative, collected by centrifugation, and then further fixed at 4 °C for 1 h. Cells were then washed in a 0.1 M cacodylate buffer and transferred to flat-bed embedding capsules, before incubation with 1% osmium tetroxide in 0.1 M cacodylate buffer for 1 h. Cells were water-washed and then dehydrated using a series of ethanol solutions: 50, 70, 90, and 100% ethanol and a transitional solution, 100% propylene oxide. Cells were then infiltrated with an epoxy resin–propylene oxide mix (1:1) overnight. The following day, the samples were infiltrated with epoxy resin for 3 × 2 h and then embedded and polymerized by heating at 60 °C for 48 h. Ultra-thin samples (80 nm) of the cells were sectioned with a diamond knife on a Leica EM UC6 ultramicrotome, mounted on 200 mesh copper grids, and then analysed using a Tecnai G2 BioTWIN TEM (FEI company, Eindhoven, The Netherlands).

### 2.2. Thiazolyl Blue Tetrazolium Bromide (MTT) Assays

Cell metabolic activity and cell viability were determined using a Thiazolyl Blue Tetrazolium Bromide (MTT) (M5655, Sigma, Poole, UK) assay, as described previously [51]. SHSY-5Y cells were seeded at 3 × 10^4^ cells/well in 96-well plates with growth medium (10% FBS). After 24 h, undifferentiated cells were exposed to ethanol (0–200 mM) diluted in growth media (10% FBS). Differentiated cells were prepared as described above and then treated with ethanol (0–200 mM) diluted in differentiation medium supplemented with 20 ng/mL BDNF. After incubation, spent medium was removed and then replaced with medium containing 10% 5 mg/mL MTT and incubated for 4 h. Plate wells which only received 10% MTT and respective growth medium served as background controls. The generated formazan crystals were suspended in a 1:1 dimethyl sulphoxide (DMSO, D8418, Sigma, Poole, UK)–isopropanol (279544, Sigma, Poole, UK) solution. The absorbance of wells was then read at 570 nm using a spectrophotometer (Multiskan Spectrum, Thermo Electron Corporation, Waltham, MA, USA). An average value was calculated from experiments performed in triplicate after the subtraction of blank (negative control) values. Cell viability was expressed as a percentage of survival compared with that from mock-treated cells. The inhibitor concentrations producing 50% loss of viability of cells (IC_50_ values) were obtained from the concentration–response curves and expressed as mean ± standard deviation (SD).

### 2.3. Lactate Dehydrogenase (LDH) Assays

Undifferentiated or differentiated SHSY-5Y cells were prepared as described above for the MTT assay and similarly treated with ethanol. After ethanol treatment, 50 µL of spent medium was removed and LDH activity determined using an assay kit (ab102526, Abcam, Cambridge, UK) according to the manufacturer’s guidelines. NADH standards were prepared according to the manufacturer’s protocol and were transferred into the same assay plate. Assays were performed at 450 nm using a spectrophotometer (Multiskan Spectrum, Thermo Electron Corporation, Waltham, MA, USA) in kinetic mode, with readings every 2 min at 37 °C, protected from light, for a total of 60 min. A NADH standard curve was generated and LDH activity measurements interpolated from the NADH standard curve. An average value was calculated from experiments performed in triplicate after the subtraction of blank (negative control) values.

### 2.4. Adenosine 5′-Triphosphate (ATP) Assays

Undifferentiated SH-SY5Y cells were seeded in 6-well plates (CC7682-7506, STARLAB International GmbH, Hamburg, Germany) at a density of 1 × 10^6^ cells/well for analysis. For differentiated cells, cells were seeded at 5 × 10^4^ cells/well in PDL-coated 6-well plates, with the differentiation protocol followed for 7 days, as described above. Cells were treated with ethanol, as before, and ATP levels were quantified using an ATP luminescence assay kit (ATP Bioluminescence Assay Kit CLS II (11 699 695 001, Roche, Germany), as per the manufacturer’s protocol. The ATP content in control and ethanol-treated samples was interpolated from an ATP standard curve, as described previously [52]. Average values were calculated from experiments performed in triplicate after the subtraction of blank (negative control) values.

### 2.5. Measurements of Reactive Oxygen Species

The generation of reactive oxygen species (ROS) was quantified using a 2′,7′-dichlorofluorescin diacetate (DCFDA) (D6883, Sigma, Poole, UK) assay. SHSY-5Y cells were seeded at 3 × 10^4^ cells/well in clear-bottom black 96-well plates (165305, Thermo Fisher Scientific, Rochester, UK) with growth medium (at 10% FBS). After 24 h, undifferentiated cells were exposed to ethanol (0–200 mM) diluted in growth medium (10% FBS) and differentiated cells were prepared as described above and then treated with ethanol (0–200 mM) diluted in differentiation medium supplemented with 20 ng/mL BDNF and 10 µM RA. Cells were treated with ethanol for 3, 6, 12, or 24 h, with 50 µM DCFDA included for the experiment duration. Cells were washed twice with ice-cold PBS and then their fluorescence was quantified using a Varioskan™ LUX multimode microplate reader (Thermo Fisher Scientific, Waltham, MA, USA) at excitation and emission spectra of 495 nm and 529 nm, respectively. Hydrogen peroxide (0.5 mM) was used as a positive control for ROS, set as 100% fluorescence [46,53]. Three to six replicate assays were performed for all data points, from which an average was calculated.

### 2.6. Cell Lysis

After ethanol or vehicle treatment of undifferentiated or differentiated SH-SY5Y cells, cells were washed with cold phosphate-buffered saline (PBS) (10010015, Life Technologies, Paisley, UK) before addition of 0.5 mL of radioimmunoprecipitation assay (RIPA, 20-188, Millipore, Burlington, MA, USA) buffer containing protease inhibitors (04693124001, mini-protease inhibitor cocktail, Sigma, Poole, UK) and a phosphatase inhibitor cocktail (P0044, Sigma, Poole, UK), with flask agitation on ice for 5 min. Cells were then scraped into the RIPA buffer, vortexed thoroughly, and then homogenized by passage through a 28 g needle 25 times. Homogenates were stored at −20 °C until required.

### 2.7. Protein Quantification

The quantitation of protein concentration was performed based on the Lowry assay [54]. Bovine serum albumin (BSA) protein was used as a protein standard. The modified Lowry assay was performed in 96-well plates using protein standard amounts of 1.25, 2.5, 5, 7.5, and 10 µg of protein. For a volume of 40 µL of cell lysates or protein standards, 20 µL of Reagent A was added followed by 160 µL of Reagent B. After 15 min, spectrophotometric measurements were taken at 740 nm using a Spectramax plate reader (Multiskan Spectrum, Thermo Electron Corporation, Waltham, MA, USA). Protein amounts of unknowns were interpolated from the BSA standard curve.

### 2.8. Determination of Protein Carbonyl Content

Undifferentiated or differentiated SH-SY5Y cells were grown to 80% confluence and then treated with ethanol for 3, 6, 12, or 24 h, as described above. After alcohol treatment, cells were washed with ice-cold PBS three times and then solubilized and lysed with RIPA buffer containing protease and phosphatase inhibitors (according to Section 2.5). Samples were vortexed for 30 s and then sonicated for 15 min on ice-cold water. Samples were then spun at 500× *g* for 10 min at 4 °C, and the supernatant was retained and centrifuged at 23,100× *g* for 40 min at 4 °C to prepare a crude cytosolic fraction [24]. Protein concentration was determined using a modified Lowry assay (according to Section 2.6) and then adjusted to 1 mg/mL for cells or brain tissue homogenates (refer to Section 2.9). An equivalent volume of 10 mM 2,4-dinitrophenylhydrazine (DNPH) (Sigma-Aldrich, Poole, UK) prepared in 2 N HCL (231-5957, Scientific Laboratory Suppliers, Nottingham, UK) was added to samples or blanks and vortex mixed, and then, samples were left in the dark for 1 h at room temperature, with vortex mixing every 10 min. Protein precipitation was initiated by the addition of an equivalent volume of ice-cold 20% (*w*/*v*) trichloroacetic acid (TCA) (Sigma-Aldrich, Poole, UK) and the samples were retained on ice for 15 min. The precipitate was washed according to a previously published method [46], before solubilization in 6 M guanidine hydrochloride (50950, Fluka Chemie AG, Buchs, Switzerland) in 50 mM phosphate buffer, pH 2.3, with incubation at 37 °C for 30 min and with vortex mixing. The protein carbonyl content (PCC) was then determined spectrophotometrically (Thermo Fisher Scientific, Fluoroskan Ascent FC, Waltham, MA, USA) at 366 nm using a molar absorption coefficient of 22,000 M^−1^cm^−1^ after subtraction of blanks. Data points were generated from assays performed in triplicate, from which an average was calculated.

### 2.9. Western Oxy-Blotting

Immuno-blotting for reactive carbonyl groups (oxidatively damaged proteins) was undertaken using an OxyBlot Protein Oxidation Detection Kit (S7150, Millipore, Burlington, MA, USA) as recommended by the manufacturer. Cytosolic protein concentrations were quantified as detailed above using a modified Lowry assay (Section 2.6). Proteins were then prepared to a concentration of 2 mg/mL via the addition of 12% sodium dodecyl sulphate (SDS) and 2,4-dinitriophenylhydrazine (DNPH) solution, and carbonyl groups were derivatized by incubation at room temperature for 15 min. Neutralization solution and then β-mercaptoethanol were added to the sample mixture, and then the proteins were resolved using Novex NuPAGE 10% Bis–Tris gel (Thermofisher Scientific, Rochester, UK) in an Xcell surelock mini-cell system with (3-N-morpholino)propanesulphonic acid (MOPS) running buffer (Thermofisher scientific, Rochester, UK), as described previously [55]. Gel proteins were transferred in a BioRad mini trans-blot cell to polyvinylene difluoride (PVDF) (Millipore, USA) membranes and probed with a rabbit anti-DNP primary antibody, followed by a goat anti-rabbit IgG (horseradish peroxidase (HRP)-conjugated) secondary antibody, as described previously [46]. Immunoreactivity was detected using a ChemiDoc MP imager (BioRad, Hertfordshire, UK), with light captured with an autoexposure setting to ensure signal linearity.

### 2.10. Human Brain Samples

The human brain samples used in this study were used in accordance with the Human Tissue Act (2004) (UK) and were supplied by the Neuropsychopharmacology Research Group from the Department of Pharmacology of the University of the Basque Country (UPV/EHU) (https://www.ehu.eus/en/web/neuropsicofarmacologia/home, accessed on 8 April 2024). Brain tissue collection was conducted in compliance with the research policies and ethical review boards for post-mortem brain studies (Basque Institute of Legal Medicine, Bilbao, Spain) and registered in the National Biobank Register of the Spanish Health Department with the study number C.0000035 (https://biobancos.isciii.es/ListadoColecciones.aspx, accessed on 8 April 2024). Diagnosis of alcoholism was carried out according to the *Diagnostic and Statistical Manual of Mental Disorders* (DSM-III-R, DSM-IV, or DSM-IV-TR; American Psychiatric Association) or International Classification of Diseases criteria (ICD-10; World Health Organization). All diagnoses were established by clinicians in charge of the patients prior to death. Six control brain samples were used, matched by age and sex to 6 alcoholic subjects, as detailed in previous studies [24,25] (Supplementary Table S1). Toxicological screening of the blood (quantitative assays for antidepressants, antipsychotics, other psychotropic drugs, and ethanol) was performed at the National Institute of Toxicology, Madrid, Spain. The brain samples used were all from the prefrontal cortex (Brodmann’s area 9) (BA 9), macroscopically dissected at the time of autopsy and stored at −80 °C until required.

### 2.11. Statistical Analysis

Data for cell viability and ATP assays are presented as means ± standard error of the mean (SEM). Statistical analysis was performed using GraphPad Prism 9.2.0 (GraphPad Prism, San Diego, CA, USA). Concentration–response curves were plotted using a non-linear regression curve fit model as line of best fit. To assess differences between control and treatment groups, one-way analysis of variance (ANOVA) or two-way ANOVA with Dennett’s multiple comparison test and Tukey’s multiple comparisons, respectively, were performed. Results were considered significant at a *p*-value below 0.05.

## 3. Results

### 3.1. Alcohol Effects on Cell Viability

**Figure 1:**
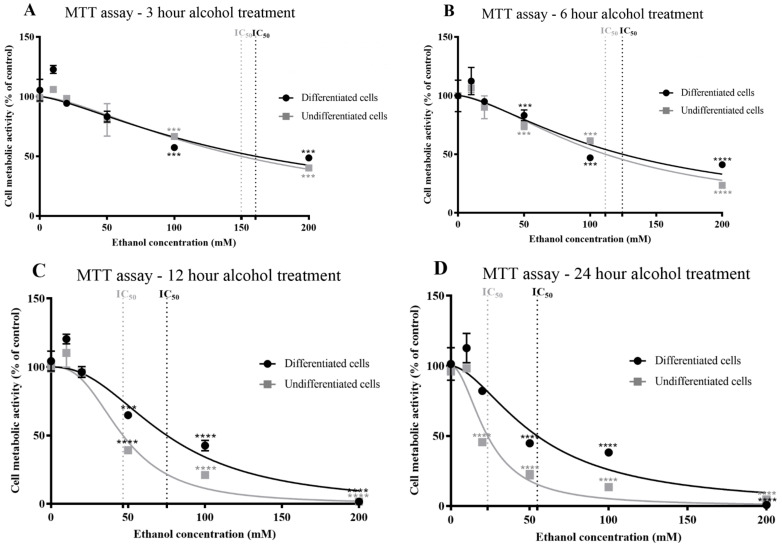
Effects of alcohol on cellular metabolic activity and viability, determined using an MTT assay. Undifferentiated or differentiated SH-SY5Y cells were exposed to alcohol (0–200 mM) for durations of 3 (**A**), 6 (**B**), 12 (**C**), and 24 (**D**) h and the levels of metabolic activity and cell viability were quantified using an MTT assay. Each data point represents the mean of at least 5 individual experiments. Marked significance: *** = *p*-value < 0.001, **** = *p*-value < 0.0001.

Undifferentiated and differentiated SH-SY5Y cells were exposed to alcohol at concentrations of 0–200 mM for 3, 6, 12, or 24 h and cell metabolic activity and viability were quantified using an MTT assay (Figure 1A–D). Alcohol reduced cell metabolic activity and viability in a concentration- and exposure duration-dependent manner from a threshold of ≥20 mM for both undifferentiated and differentiated SH-SY5Y cells (Figure 1A–D and Supplementary Table S2).

After 3 or 6 h alcohol exposure, cell metabolic activity for both undifferentiated or differentiated SH-SY5Y cells was similar and inversely proportional to alcohol concentration, such that there was an approximately linear decline in cell viability with increasing alcohol concentration (Figure 1A,B). After 12 or 24 h incubation with alcohol, the inhibitor–response curves showed significant reduction in cell viability at 50 mM alcohol (*p* < 0.0001) (Figure 1C,D). Differentiated cells were more resistant to alcohol toxicity than undifferentiated cells, with higher concentrations required to induce 50% inhibition of cell viability (IC_50_) (Figure 1A–D, Table 1, and Supplementary Table S3). The lowest concentration of alcohol examined (10 mM) increased cell metabolic activity, although non-significantly (*p* = 0.113), by 6–11% in differentiated cells and 1–10% in undifferentiated cells (*p* = 0.08) (Figure 1A–D).

**Figure 2:**
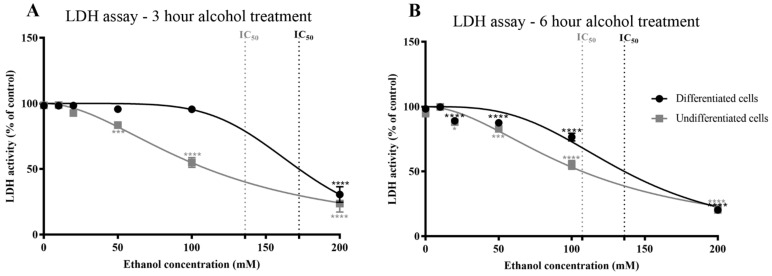

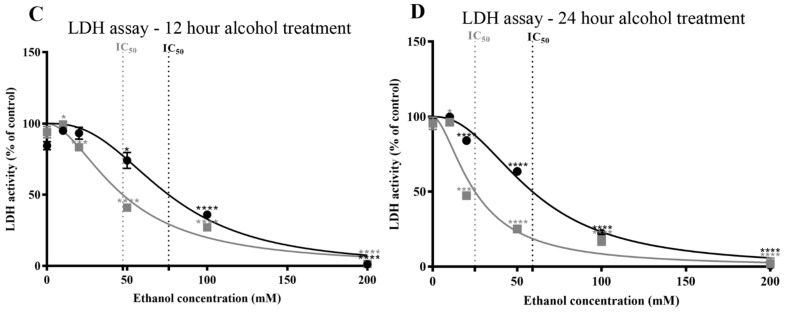
Alcohol effects on cell viability determined using an LDH activity assay. Undifferentiated or differentiated SH-SY5Y cells were exposed to alcohol (0–200 mM) for durations of 3 (**A**), 6 (**B**), 12 (**C**), and 24 (**D**) h, and the activity of extracellular LDH was quantified. Each data point represents the mean of at least 5 individual experiments. Marked significance: * = *p*-value < 0.05, *** = *p*-value < 0.001, **** = *p*-value < 0.0001.

Since MTT assays provide insight into cell metabolic activity and this may not always correlate with cell viability, the liberation of extracellular LDH was used as an independent method for the determination of cell viability in response to alcohol. Similar to the MTT assays, undifferentiated and differentiated cell viability decreased in proportion to the alcohol concentration and length of exposure time (Figure 2A–D and Supplementary Table S4). The threshold for a significant reduction of cell viability was a concentration of alcohol of ≥20 mM for 6 h exposure time (*p* < 0.001 for undifferentiated cells and *p* < 0.0001 for differentiated cells) (Figure 2B). Non-linear regression analysis showed that undifferentiated cells were more sensitive to the toxic effects of alcohol, with lower IC_50_ concentrations, in keeping with the MTT data (Table 1 and Supplementary Table S2).

**Figure 3:**
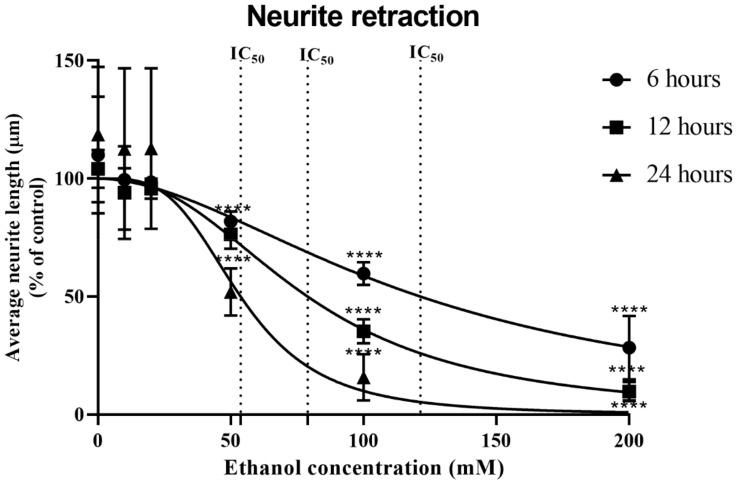
Neurite retraction in response to alcohol treatment. Differentiated SHSY-5Y cells were treated with alcohol over a concentration range of 0–200 mM for 6–24 h and the length of neuritic projections was quantified. Experiments were conducted in triplicate and each data point represents the mean of at least 5 individual experiments (±SD), with vehicle control experiments set at 100%. Significant reductions in neuritic projections were observed at 50 mM alcohol for all time points. Marked significance: **** = *p*-value < 0.0001.

Additionally, the alcohol-induced reduction in cell viability and influence on neuritic projections (Figure 3) were assessed via direct observation of the cells and photographic image capture (Supplementary Figure S1 and Supplementary Table S5). Alcohol triggered a significant reduction in neuritic arborization from a threshold concentration of 50 mM for 6 (*p* < 0.001), 12 (*p* < 0.001), and 24 h (*p* < 0.001) exposures (Figure 3 and Supplementary Table S5).

### 3.2. Alcohol Effects on Cellular Bioenergetics and the Liberation of Reactive Oxygen Species

**Figure 4:**
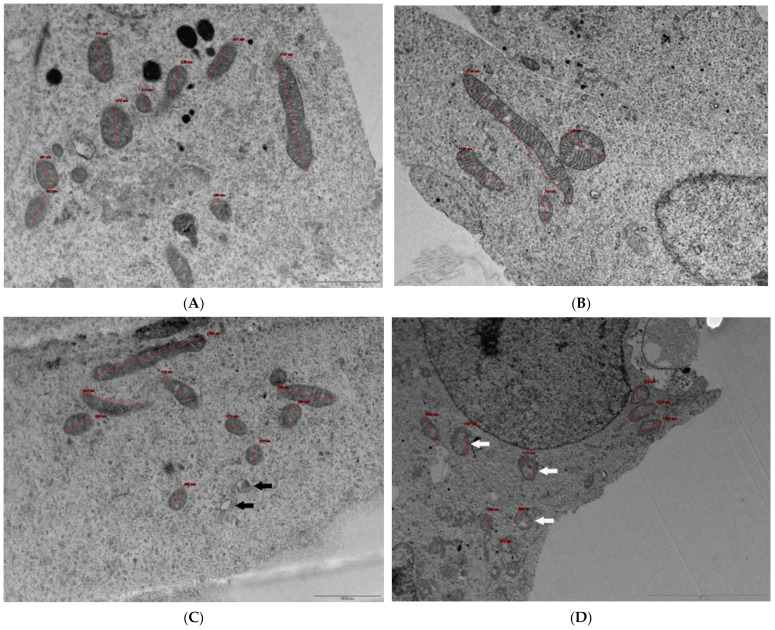
TEM images of control and alcohol-treated cells. (**A**) ×16,500 magnification of control (untreated) cells. Mitochondria are clear with well-visible cristae, some of which have been sized (in red) for reference. Scale bar: 1000 nm. (**B**) ×9900 magnification of cells treated with 50 mM ethanol for 24 h. Mitochondria are distinguishable with visible cristae that are patchy in places, and some elongated mitochondria can be observed. Mitochondrion measurements have been included (in red) for reference. Scale bar: 2000 nm. (**C**) ×16,500 magnification of cells treated with 100 mM ethanol for 24 h. Mitochondria are distinguishable with visible cristae that are patchy in places, some elongated mitochondria are visible, as well as some vacuolar regions perhaps generated from mitophagy (examples indicated with black arrows). Mitochondrion measurements (in red) have been included for reference. Scale bar: 1000 nm. (**D**) ×6000 magnification of cells treated with 200 mM ethanol for 24 h. Mitochondria are distinguishable with some visible cristae but clear regions within mitochondria (examples indicated with white arrows) and some vacuolar regions presumed to be generated from mitophagy. Mitochondrion measurements (in red) have been included for reference. Scale bar: 5000 nm. For TEM transverse section images, up to 19 fields of view were analysed, with random unbiased selection. Images were captured using a MegaView SIS camera, with representative images included.

Direct effects on mitochondrial morphology were examined using transmission electron microscopy (TEM) (Figure 4A–D). Alcohol at concentrations of ≥50 mM resulted in more translucent mitochondria (less electron dense) and some vacuoles were present within cells, which may reflect mitophagy.

**Figure 5:**
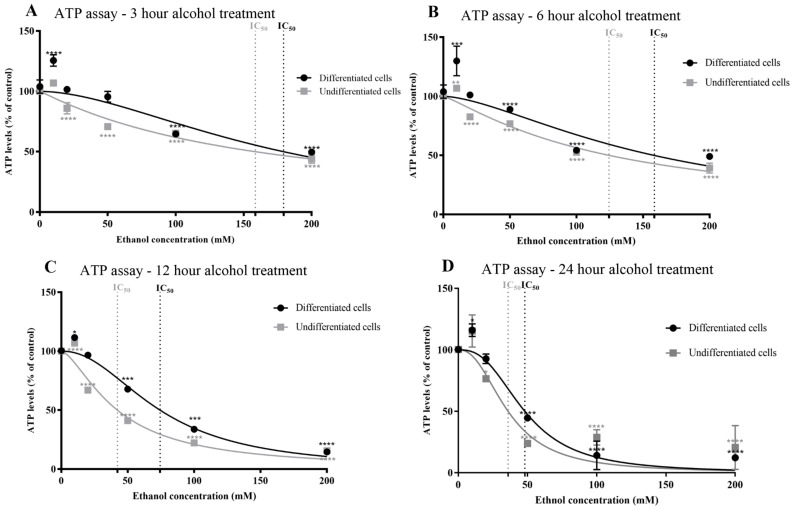
Effects of alcohol on cellular ATP levels determined using an ATP bioluminescence assay. Undifferentiated or differentiated SH-SY5Y cells were exposed to alcohol (0–200 mM) for durations of 3 (**A**), 6 (**B**), 12 (**C**), and 24 (**D**) h, and the level of cellular ATP was quantified using an ATP bioluminescence assay. Each data point represents the mean of at least 5 individual experiments. Marked significance: * = *p*-value < 0.1, ** = *p*-value < 0.01, *** = *p*-value < 0.001, **** = *p*-value < 0.0001.

The effect of alcohol on cellular bioenergetic capacity was determined via quantitation of ATP levels. An alcohol-induced decline in ATP levels was observed which correlated with alcohol concentration and exposure duration and mirrored the MTT alcohol response curves for both undifferentiated and differentiated SH-SY5Y cells (Figure 5A–D, and Supplementary Table S6), with similar IC_50_ values (refer to Table 1 and Supplementary Table S2). A significant reduction in ATP levels was evident from an exposure concentration of ≥20 mM and 3 h exposure for undifferentiated cells (*p* < 0.0001). Interestingly, the induction of cell metabolic activity (MTT assay results) observed after 10 mM alcohol exposure was reiterated for ATP production. The resistance of differentiated cells to alcohol was also evident from measurements of ATP levels, such that a significant reduction in ATP was observed with alcohol concentrations of ≥50 mM and application of at least 6 h (*p* = 0.0294).

**Figure 6:**
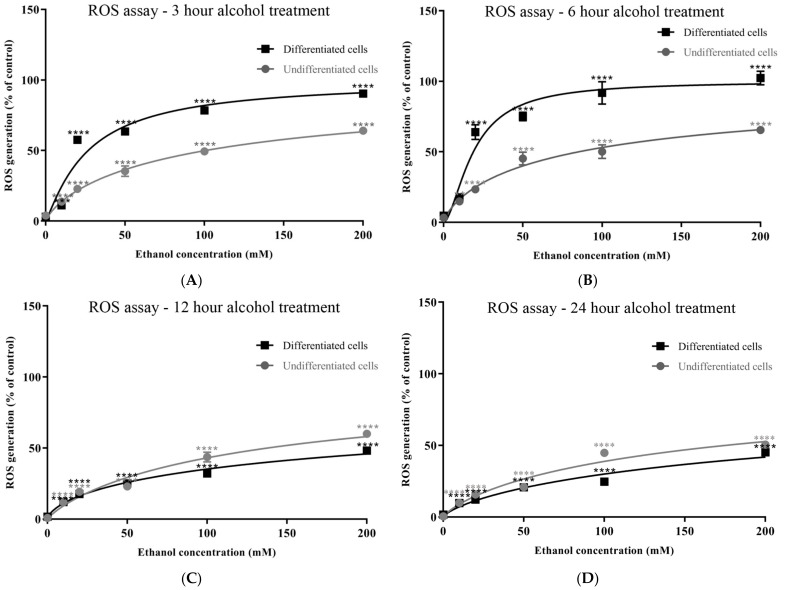
Alcohol induction of ROS levels determined using a DCFDA assay. Undifferentiated or differentiated SH-SY5Y cells were exposed to alcohol (0–200 mM) for durations of 3 (**A**), 6 (**B**), 12 (**C**), and 24 (**D**) h, and the levels of cellular ROS were quantified relative to that induced by H_2_O_2_. Each data point represents the mean of at least 5 individual experiments. Marked significance: ** = *p*-value < 0.01, *** = *p*-value < 0.001, **** = *p*-value < 0.0001.

The production of reactive oxygen species (ROS) was monitored over the 3–24 h time course through measuring the oxidation of 2′,7′-dichlorodihydrofluorescein in a DCFDA assay. ROS levels increased in proportion to alcohol concentrations at all time points, with ROS levels that peaked at 3 and 6 h (Figure 6A–D and Supplementary Table S7). Differentiated cells were notably more potent producers of ROS than undifferentiated cells, with significantly higher levels of ROS liberated after 20 and 50 mM alcohol exposures at the 3 and 6 h time points (*p* < 0.0001) (Figure 6A–D and Supplementary Table S3).

**Figure 7:**
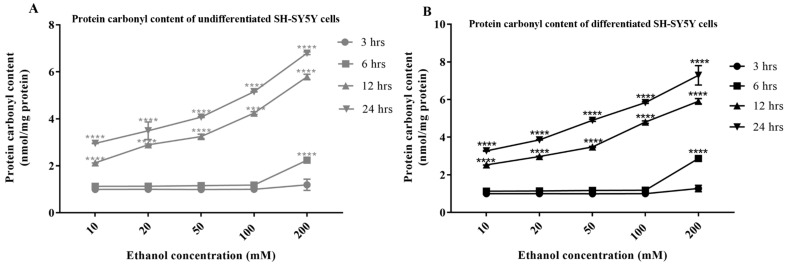
Alcohol induction of protein carbonyl content. Undifferentiated (**A**) or differentiated SH-SY5Y cells (**B**) were exposed to alcohol (0–200 mM) for durations of 3, 6, 12, and 24 h, and the levels of protein carbonyl content (PCC) were quantified via spectrophotometry. Each data point represents the mean of at least five individual experiments. For marked significance: **** = *p*-value < 0.0001.

In line with the production of cellular ROS, the level and time course of production of oxidatively damaged proteins was quantified via determination of total protein carbonyl content (PCC). PCC increased in undifferentiated and differentiated SH-SY5Y cells in accordance with the concentration of alcohol; significant levels were detected from 10 mM alcohol, the lowest concentration examined (*p* < 0.0001) (Figure 7A,B). However, there was a delay in the accumulation of PCC, with significant increases above baseline (≈1 nmol/mg of protein) detected after 12 or 24 h, and this had a higher positive correlation with ROS levels (Supplementary Table S8). PCC profiles were similar for undifferentiated cells and differentiated cells but with statistically higher levels in differentiated cells from a threshold concentration of 50 mM and 12 h alcohol exposure (*p* < 0.0001) (Supplementary Table S3).

**Figure 8:**
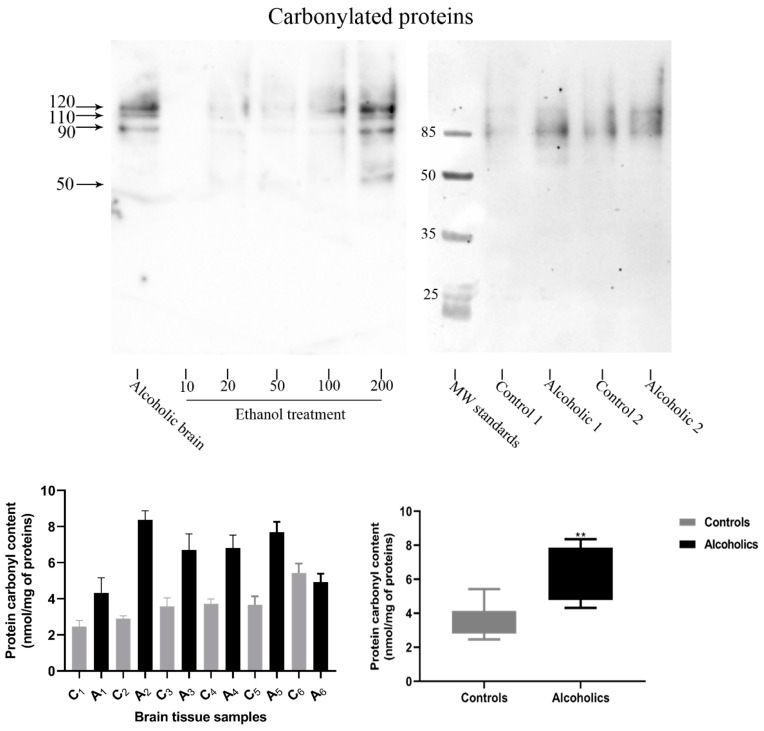
Quantitation and characterization of carbonylated proteins. Differentiated SH-SY5Y cells were exposed to alcohol (10–200 mM) for 24 h and carbonylated proteins were detected via oxy-blotting. Major carbonylated proteins were detected at 120, 110, 90, and 50 kDa in cells and control or alcoholic brain tissue (upper panel). Protein carbonylated content of proteins from six control and six matched alcoholic brain tissue samples were quantified via spectrophotometry (lower panels). Each data point or blotting image is a representation of at least 3 individual experiments. Significance: ** = *p*-value < 0.01.

In order to characterise the carbonylated proteins, oxy-blotting was performed. Carbonylation (protein oxidation) was detected in several proteins, at denatured molecular weights of 120, 110, 90, and 50 kDa and with levels that increased in accordance with alcohol concentration (Figure 8). The profile of carbonylated proteins was similar to that detected in the brains of alcoholic subjects and, to a lesser extent, age- and sex-matched controls (Figure 8). Total PCC was increased in alcoholic brains compared with those of control subjects, with levels of approximately 4–8 nmols/mg of protein in the alcoholic brain samples, similar to those detected after the highest acute alcohol treatment of cells (Figure 8).

## 4. Discussion

Alcohol has toxic effects on the brain that may be particularly detrimental during periods of neurogenesis and differentiation, such as those experienced during neurodevelopment. To consider this further, including the potential involvement of redox stress, a comparison of alcohol neurotoxicity was undertaken between undifferentiated neuroblastoma cells and those that had been acutely differentiated into a neuronal phenotype. Cytotoxicity assessment using MTT and LDH assays showed that differentiation rendered cells more resistant to alcohol, with higher alcohol concentrations required to reduce cell viability. However, somewhat in contrast to alcohol’s effects on cell viability, the levels of ROS and corresponding production of carbonylated (oxidatively damaged) proteins were more extensive in differentiated cells. The characterization of carbonylated proteins revealed proteins with denatured molecular weights that overlapped with those present within the brains of alcoholic subjects, and further, PCC increased in alcoholics compared with matched controls. Hence, cell differentiation may promote resistance to alcohol-induced death but render cells more susceptible to the accumulation of oxidatively damaged proteins.

We chose to model alcohol neurotoxicity using SH-SY5Y cells due to their human origin, broad application for neurotoxicity studies, and potential for manipulation to cell cycle-synchronized, trophic-dependent, differentiated cells that display morphology, neuritic arborization, and protein expression indicative of neurons [46,47,56,57,58,59] (Supplementary Figure S1). We assessed the cytotoxicity of alcohol using MTT and LDH assays, as well as through visual inspection of cells to confirm reduced viability (Figure 1, Figure 2 and Figure 3, and Supplementary Figure S1). Cell viability using MTT assays primarily relies on the activity of oxidoreductase and dehydrogenase enzymes in healthy (metabolically active) cells [60]. However, relatively low concentrations of agents such as phytochemicals can induce cell metabolic activity, with optical density readings that exceed those of control values [52], and this was observed after incubation with 10 mM alcohol (Figure 1). We therefore undertook another independent method for the quantification of changes in cell viability, using the liberation of extracellular LDH due to loss of membrane integrity [61]. Both methods generated similar IC_50_ values for undifferentiated or differentiated cells to those from MTT assays. Surprisingly, IC_50_ values were higher for differentiated cells (Table 1), indicative that differentiation was protective against alcohol. This contrasts with the effects of some toxic agents, such as organophosphate and carbamate pesticides, which are more toxic to differentiated SH-SY5Y cells [46] but not to other neurotoxicants, such as 1-methyl-4-phenyl-1,2,3,6-tetrahydropyridine (MPTP) [62], an agent that can induce Parkinsonian phenotypes in animals [63,64].

Cell viability experiments were undertaken across a broad concentration range of 10–200 mM alcohol for 3–24 h. This starting point for cell toxicity assays reflected blood alcohol concentrations (BACs) of 0.04–0.05% (≈9–11 mM) that represent a central nervous system (CNS) threshold for impact on psychomotor tasks [65]. The exposure to 20 to 50 mM alcohol corresponds to BACs that can arise from the consumption of several alcoholic beverages in a period of a few hours, concentrations consistent with intoxication for susceptible individuals [65]. The very high concentrations of 100 and 200 mM alcohol that were assessed can induce loss of consciousness, coma, or even death, although patients with alcohol use disorders (AUDs) often develop tolerance to alcohol’s CNS effects as well as displaying heightened alcohol metabolism enabling them to withstand such high systemic BACs (>100 mM) and still may not display signs of intoxication [66].

Alcohol can damage and alter the morphology of mitochondria and promote the liberation of ROS [67,68]. We therefore investigated the ability of alcohol to affect cellular ATP levels and the production of ROS. The time course of ATP decline in response to alcohol mirrored the concentration–response curves observed from MTT and LDH assays, consistent with a shutdown of ATP production and loss of cell viability [69]. The opacity of the inner mitochondrial regions was reduced after exposure to the higher alcohol concentrations, with mitochondria observed as less electron-dense within the cristae (Figure 4), and this may correlate with a lowered ability to synthesize ATP [70]. Additionally, the capacity to produce ATP is reduced if sufficient mitochondria are damaged to trigger mitophagy and their removal, and at higher concentrations of alcohol exposure, more vacuoles were evident, which may have arisen from ongoing mitophagy (Figure 4).

Alcohol exposure induced ROS and increased the levels of oxidatively damaged proteins. Relatively low levels of ROS can impact cellular signalling pathways and may be functional, but there is a threshold at which ROS levels are detrimental to the cell and induce apoptosis [71]. Our studies show that alcohol induced ROS production and increased protein carbonyl content at the lowest levels of alcohol exposure examined (10 mM) (Figure 6), and for these exposures, there was no reduction in cell viability (Figure 1 and Figure 2). By contrast, higher alcohol concentrations (and exposure durations) increased ROS production and reduced cell viability, in keeping with the ability of alcohol to induce apoptotic cell death [40,41,42,43]. From alcohol exposures of 20 mM, immuno-blotting provided a means to characterise the major proteins that were oxidatively damaged; it was noteworthy that the proteins that accumulated oxidative damage after exposure to alcohol in vitro mirrored those observed in brain tissue from control and alcoholic patients. This suggests that there is a subset of cellular proteins that are particularly vulnerable to oxidative damage.

The endogenous levels of oxidative damage in alcoholic brains were similar to those from the highest induction of cellular toxicity in vitro (100–200 mM alcohol exposure) and were higher than those from age- and sex-matched control subjects (Figure 8). The molecular weights of these proteins (120, 100, 90, and 50 kDa) were found to be similar to those that accumulated in SH-SY5Y cells in response to exposures to organophosphate and carbamate pesticides [46], and this presumably reflects their relative abundance and vulnerability to oxidation. We have postulated that these protein bands may include MAP-tau and tubulin (90 and 50 kDa, respectively) due to their increased expression during differentiation [46], but the identity of these proteins, and how oxidative damage could influence protein function, will need to be addressed in future studies.

## 5. Conclusions

Our results show that newly differentiated neuronal cells are, surprisingly, more resistant to cell death from alcohol than undifferentiated cells. However, for similar levels of alcohol exposure, alcohol induced higher levels of ROS and the formation of oxidatively damaged proteins in newly differentiated cells. Neuritic arborization was blunted and neuronal cells were killed after 6 and 12 h exposure to ≥50 mM alcohol. Such levels of alcohol would correspond to exposure likely to be experienced only through sustained excessive drinking. Importantly, our experiments were limited since we could not take into account reduced alcohol concentrations due to metabolism. Our in vitro study was also limited in its capacity to reproduce the complexity of the multiple interacting cell types in vivo, since only a single population of neuronal cells was examined. Furthermore, brain tissue exhibits regional damage to alcohol [29,31,32,33] which may reflect differences in vulnerability between cell types, and our model may not be representative of other cell types. Nevertheless, a benefit of our approach is that the cells employed were homogenous, facilitating the generation of controlled experiments and reproducible and robust experimental data.

Since the lowest concentrations of alcohol examined (10 mM) can still induce the production of ROS and increase the levels of carbonylated proteins, depending on the turnover of these proteins, they could persist and impact neuronal cell function. Hence, the reduced cognitive capacity that arises in FASD [38,39,40] or that experienced by chronic heavy drinkers [9,11,14] could reflect both a reduction in numbers of neurons and the cellular damage to and limited functional capacity of surviving neurons. This raises the possibility that countering the induction of oxidative stress, such as through enhancement of the cellular antioxidant capacity, could have benefits for acute and possibly chronic alcohol exposure through reducing the potential for neuronal loss and accrued oxidative damage.

## Figures and Tables

**Table 1:** Toxicity of alcohol to undifferentiated and differentiated SHSY-5Y cells.

Cell Type	Treatment Duration (Hours)	MTT Assay		LDH Assay		ATP Assay	
		IC_50_	R^2^	IC_50_	R^2^	IC_50_	R^2^
Undifferentiated	3	149.8 ± 18.6	0.8800	110.6 ± 3.1	0.9878	158.5 ± 17.3	0.9149
Differentiated		160.5 ± 25.8	0.7969	172.5 ± 3.4	0.9863	179.4 ± 26.3	0.7732
Undifferentiated	6	111.5 ± 7.6	0.9453	107.2 ± 4.6	0.9722	124.4 ± 10.6	0.9430
Differentiated		124.4 ± 14.7	0.8573	136.1 ± 5.9	0.9507	158.2 ± 29.2	0.7196
Undifferentiated	12	46.7 ± 3.1	0.9648	46.9 ± 3.1	0.9743	42.2 ± 3.9	0.9445
Differentiated		75.25 ± 7.0	0.9268	75.8 ± 4.5	0.9551	74.4 ± 3.7	0.9745
Undifferentiated	24	23.3 ± 2.1	0.9372	24.9 ± 2.374	0.9371	36.0 ± 5.1	0.8476
Differentiated		54.83 ± 6.5	0.9016	59.10 ± 2.3	0.9391	48.08 ± 3.4	0.9551

## Data Availability

Additional data that supports this work are available as Supplementary Data files.

## Supplementary Materials

The following supporting information can be downloaded at https://www.mdpi.com/article/10.3390/antiox13050580/s1, Supplementary Table S1: Demographics of the human brain samples. Supplementary Table S2: Alcohol toxicity in undifferentiated and differentiated SH-SY5Y cells measured via MTT assay. Supplementary Table S3: A comparison of the effects of alcohol on undifferentiated vs. differentiated SHSY-5Y cells. Supplementary Table S4: Alcohol toxicity to undifferentiated and differentiated SH-SY5Y cells measured via LDH assay. Supplementary Table S5: Neurite reduction in response to alcohol treatment. Supplementary Table S6: Alcohol toxicity in undifferentiated and differentiated SH-SY5Y cells measured via LDH assay. Supplementary Table S7: Alcohol induction of ROS in undifferentiated and differentiated SH-SY5Y cells measured via DCFDA assay. Supplementary Table S8: Correlation between the levels of ROS and protein carbonyl content in response to alcohol exposure. Supplementary Figure S1: Treatment of undifferentiated and differentiated SHSY-5Y cells with alcohol. Figure S2: Samples of original Western oxy-blots.
