# Supplementary material for: Alcohol Triggers the Accumulation of Oxidatively Damaged Proteins in Neuronal Cells and Tissues
Source: Antioxidants (Basel). 2024 May 8;13(5):580. doi: 10.3390/antiox13050580 (PMC11117938; doi:10.3390/antiox13050580)
Supplement: Supplementary file 1 [file antioxidants-13-00580-s001.zip › antioxidants-2981417-supplementary.pdf]

Supplementary Data.

Supplementary Table S1: Demographics of the human brain samples.

Identifier	Psychiatric diagnosis	Age (years)	Gender (F/M)	PMD (h)	Cause of death	Ethanol in blood (mg/mL)	Other drugs in blood
**C1**	None	66	M	50	Accident	0	(-)
**A1**	Alcoholism	71	M	17	Accident	0.35	(-)
**C2**	Bipolar	50	M	6	Suicide	0	Diazepam
**A2**	Alcoholism	50	M	3	Suicide	0	(-)
**C3**	Anxiety	44	M	15	Suicide	0	(-)
**A3**	Alcoholism	40	M	21	Suicide	0	(-)
**C4**	Depression	58	F	48	Suicide	Not determined	Not determined
**A4**	Alcoholism	57	F	24	Suicide	0.23	Methanol
**C5**	Anxiety	48	F	9	Suicide	0	Ephedrine
**A5**	Alcoholism	50	F	6	Suicide	0	Hydroclorothiazide
**C6**	OCD	65	M	19	Suicide	0	Nodiazepam
**A6**	Alcoholism	71	M	19	Suicide	0.55	(-)

Abbreviations: PMD, post-mortem delay.

Supplementary Table S2: Alcohol toxicity to undifferentiated and differentiated SH-SY5Y cells measured by a MTT assay.

**Cell type**	**Treatments (mM)**	**3 h vs 6 h**	**3 h vs 12 h**	**3 h vs 24 h**	**6 h vs 12 h**	**6 h vs 24 h**	**12 h vs 24 h**
Undifferentiated	10	NS	NS	NS	NS	NS	NS
	20	NS	NS	<0.0001	NS	<0.0001	<0.0001
	50	NS	<0.0001	<0.0001	<0.0001	<0.0001	0.0067
	100	NS	<0.0001	<0.0001	<0.0001	<0.0001	NS
	200	NS	<0.0001	0.0007	0.0003	0.0437	NS
Differentiated	10	NS	NS	NS	NS	NS	NS
	20	NS	NS	NS	NS	NS	NS
	50	NS	0.0085	<0.0001	0.0122	<0.0001	0.0037
	100	NS	0.0483	0.0061	NS	NS	NS
	200	NS	<0.0001	<0.0001	<0.0001	<0.0001	NS

Abbreviations: NS, non-significant (>0.05).

Supplementary Table S3: A comparison of the effects of alcohol on undifferentiated vs differentiated SHSY-5Y cells.

		**Undifferentiated vs differentiated SH-SY5Y cells**				
**Alcohol treatment (mM)**	**Exposure time point (hrs)**	**MTT assay**	**LDH assay**	**ATP assay**	**ROS assay**	**PCC assay**
**10**	3	0.0075	NS	<0.0001	NS	NS
	6	NS	NS	<0.0001	NS	NS
	12	NS	NS	0.0004	NS	<0.0001
	24	0.0161	NS	NS	NS	NS
**20**	3	NS	NS	<0.0001	<0.0001	NS
	6	NS	NS	0.0002	<0.0001	NS
	12	NS	0.0026	<0.0001	NS	NS
	24	<0.0001	<0.0001	NS	0.0016	NS
**50**	3	NS	0.0001	<0.0001	<0.0001	NS
	6	NS	NS	0.0138	<0.0001	NS
	12	<0.0001	<0.0001	<0.0001	NS	0.0035
	24	0.0002	<0.0001	0.0098	NS	0.0004
**100**	3	NS	<0.0001	NS	<0.0001	NS
	6	0.0493	<0.0001	NS	<0.0001	NS
	12	<0.0001	0.0070	<0.0001	<0.0001	<0.0001
	24	<0.0001	0.0278	NS	<0.0001	0.0028
**200**	3	NS	0.0342	NS	<0.0001	NS
	6	0.0120	NS	NS	<0.0001	<0.0001
	12	NS	NS	NS	<0.0001	NS
	24	NS	NS	NS	<0.0001	0.0391

Abbreviations: NS, non-significant (>0.05).

Supplementary Table S4: Alcohol toxicity to undifferentiated and differentiated SH-SY5Y cells measured by a LDH assay.

Cell type	Treatments (mM)	3 h vs 6 h	3 h vs 12 h	3 h vs 24 h	6 h vs 12 h	6 h vs 24 h	12 h vs 24 h
Undifferentiated	10	NS	NS	NS	NS	NS	NS
	20	NS	NS	<0.0001	NS	<0.0001	<0.0001
	50	NS	<0.0001	<0.0001	<0.0001	<0.0001	0.0007
	100	NS	<0.0001	<0.0001	<0.0001	<0.0001	NS
	200	NS	<0.0001	<0.0001	<0.0001	<0.0001	NS
Differentiated	10	NS	NS	NS	NS	NS	NS
	20	NS	NS	0.0015	NS	NS	NS
	50	NS	<0.0001	<0.0001	0.0037	<0.0001	0.0318
	100	<0.0001	<0.0001	<0.0001	<0.0001	<0.0001	0.0029
	200	0.0002	<0.0001	<0.0001	NS	NS	NS

Abbreviations: NS, non-significant (>0.05).

Supplementary Table S5: Neurite reduction in response to alcohol treatment.

Cell Type	Treatment duration (hours)	Neurite reduction	
		IC_{50}	R^2
Differentiated	3	Not observable	-
Differentiated	6	121.4 ± 7.6	0.8383
Differentiated	12	79.0 ± 3.2	0.9272
Differentiated	24	53.72 ± 7.5	0.8383

The concentration of alcohol producing 50% (IC_{50}) neurite reduction was calculated by non-linear regression.

Supplementary Table S6: Alcohol toxicity to undifferentiated and differentiated SH-SY5Y cells measured by a LDH assay.

Cell type	Treatments (mM)	3 h vs 6 h	3 h vs 12 h	3 h vs 24 h	6 h vs 12 h	6 h vs 24 h	12 h vs 24 h
Undifferentiated	10	NS	NS	NS	NS	NS	NS
	20	NS	0.0004	NS	0.0039	NS	NS
	50	NS	<0.0001	<0.0001	<0.0001	<0.0001	0.0013
	100	0.0381	<0.0001	<0.0001	<0.0001	<0.0001	0.5266
	200	NS	<0.0001	0.0019	<0.0001	0.0334	NS
Differentiated	10	NS	0.0035	NS	0.0002	NS	NS
	20	NS	NS	NS	NS	NS	NS
	50	NS	<0.0001	<0.0001	0.0003	<0.0001	<0.0001
	100	NS	<0.0001	<0.0001	<0.0001	<0.0001	0.0008
	200	NS	<0.0001	<0.0001	<0.0001	<0.0001	NS

Abbreviations: NS, non-significant (>0.05).

Supplementary Table S7: Alcohol induction of ROS in undifferentiated and differentiated SH-SY5Y cells measured by a DCFDA assay.

**Cell type**	**Treatments (mM)**	**3 h vs 6 h**	**3 h vs 12 h**	**3 h vs 24 h**	**6 h vs 12 h**	**6 h vs 24 h**	**12 h vs 24 h**
Undifferentiated	10	NS	NS	NS	NS	NS	NS
	20	NS	NS	NS	NS	NS	NS
	50	0.0297	0.0038	0.0006	<0.0001	<0.0001	NS
	100	NS	NS	NS	NS	NS	NS
	200	NS	NS	0.0058	NS	0.0004	0.0386
Differentiated	10	NS	NS	NS	NS	NS	NS
	20	<0.0001	<0.0001	NS	NS	<0.0001	<0.0001
	50	<0.0001	<0.0001	0.0160	NS	<0.0001	<0.0001
	100	<0.0001	<0.0001	0.0039	NS	<0.0001	0.0008
	200	<0.0001	<0.0001	0.0111	NS	<0.0001	<0.0001

Abbreviations: NS, non-significant (>0.05).

Supplementary Table S8: Correlation between the levels of ROS and protein carbonyl content in response to alcohol exposures.

Cell Type	Treatment duration (hours)	ROS vs PCC		
		r	R^2	***P* value**
Undifferentiated	3	0.4874	0.2375	NS
Differentiated		0.5758	0.3315	NS
Undifferentiated	6	0.7193	0.5175	NS
Differentiated		0.8584	0.7369	NS
Undifferentiated	12	0.9940	0.9881	0.0006
Differentiated		0.8624	0.7437	NS
Undifferentiated	24	0.9592	0.9200	0.0098
Differentiated		0.9579	0.9175	0.0103

Abbreviations: NS, non-significant (>0.05). r, Pearson’s correlation coefficient.

Supplementary Figure S1: Treatment of undifferentiated and differentiated SHSY-5Y cells with alcohol.

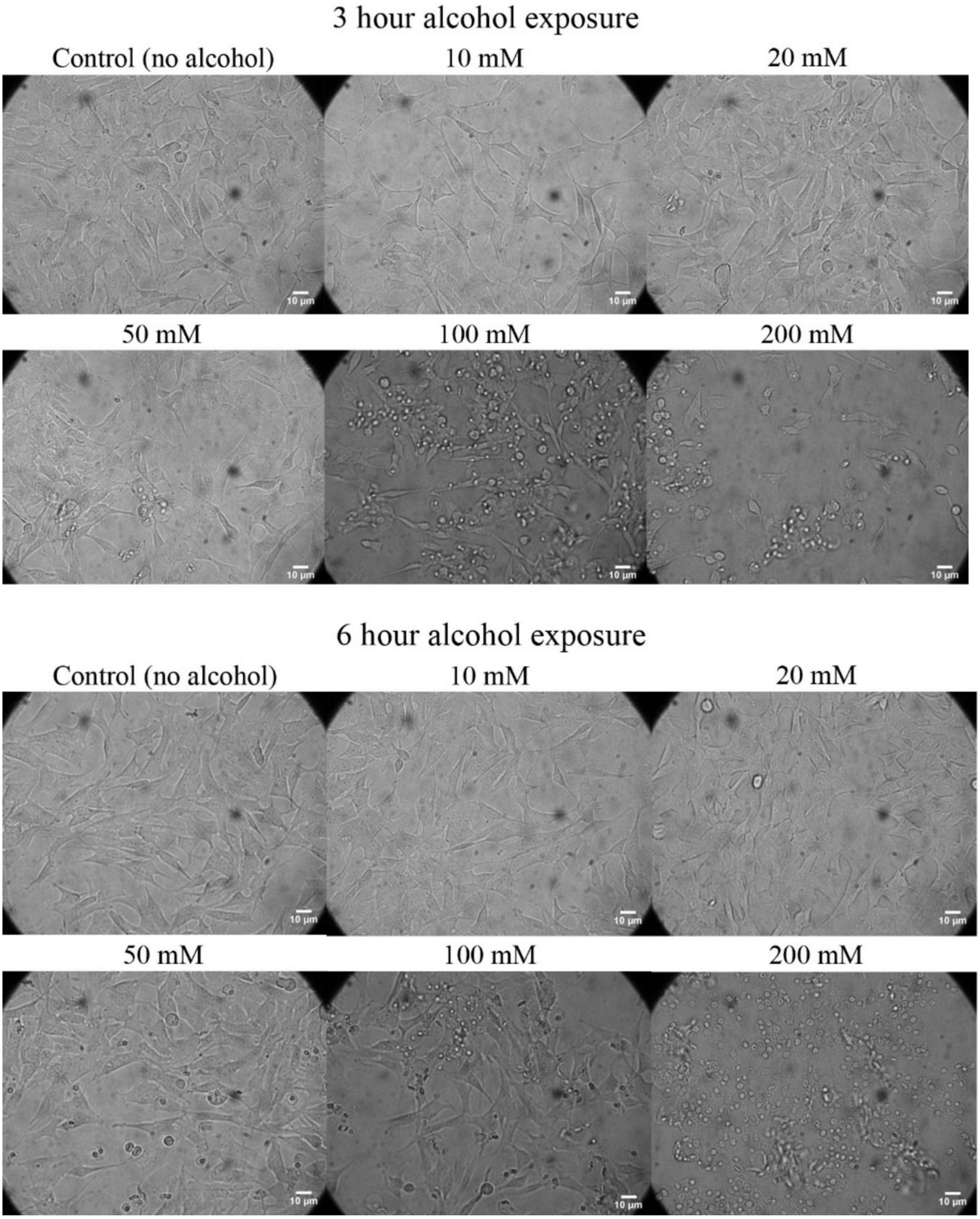

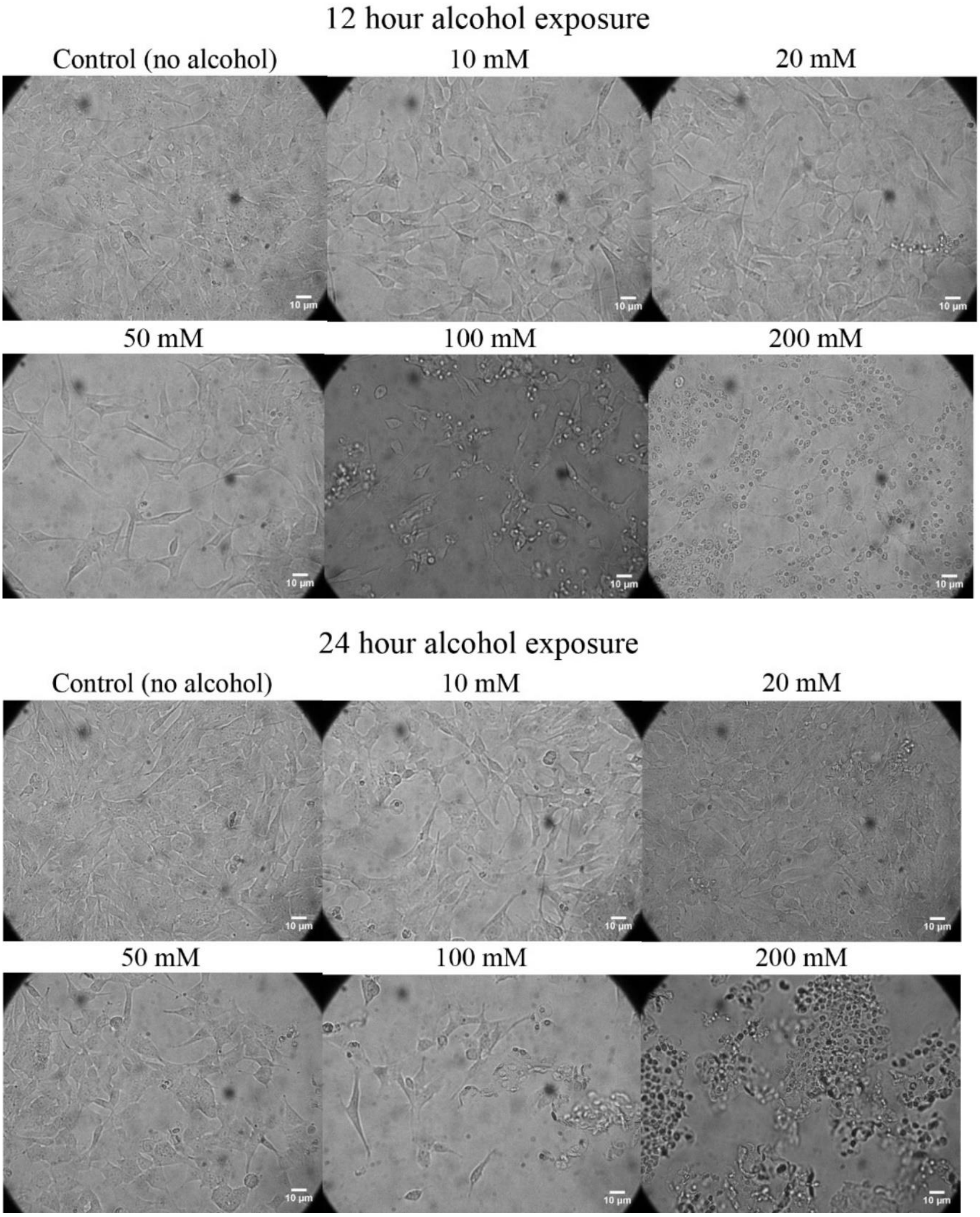

Undifferentiated SH-SY5Y cells were treated with 0–200 mM alcohol for 3, 6, 12, and 24 hours, and images captured using a phase contrast microscope. Scale bar: 10 µm.

3 hour alcohol exposure

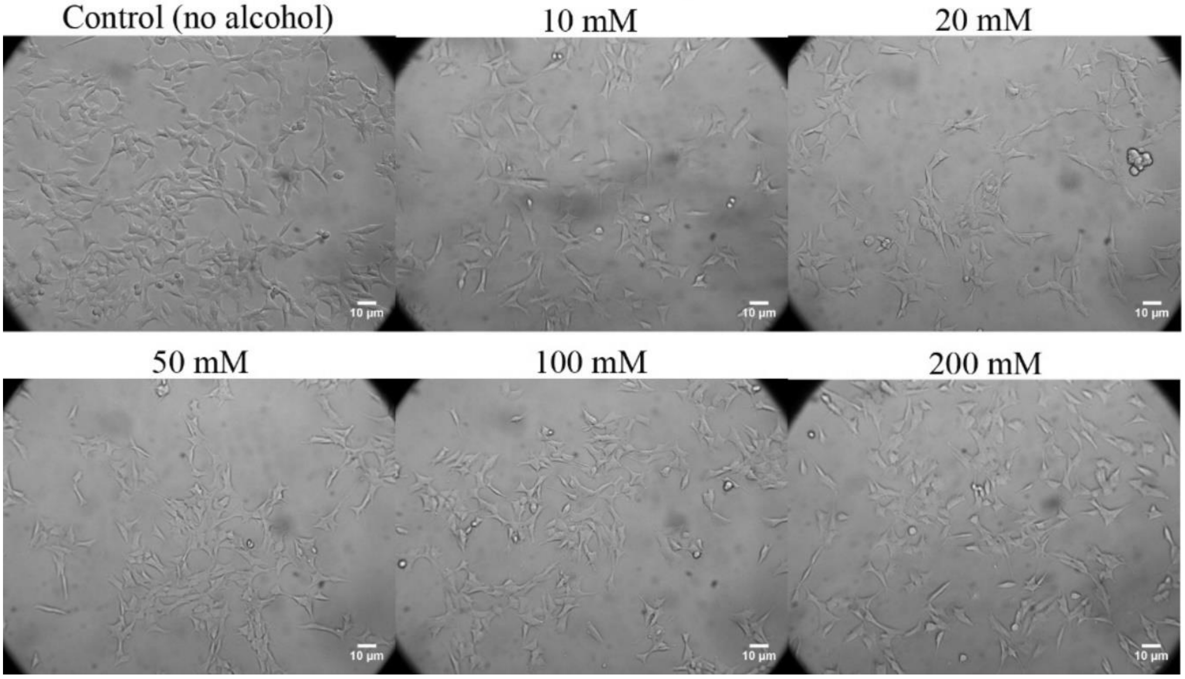

6 hour alcohol exposure

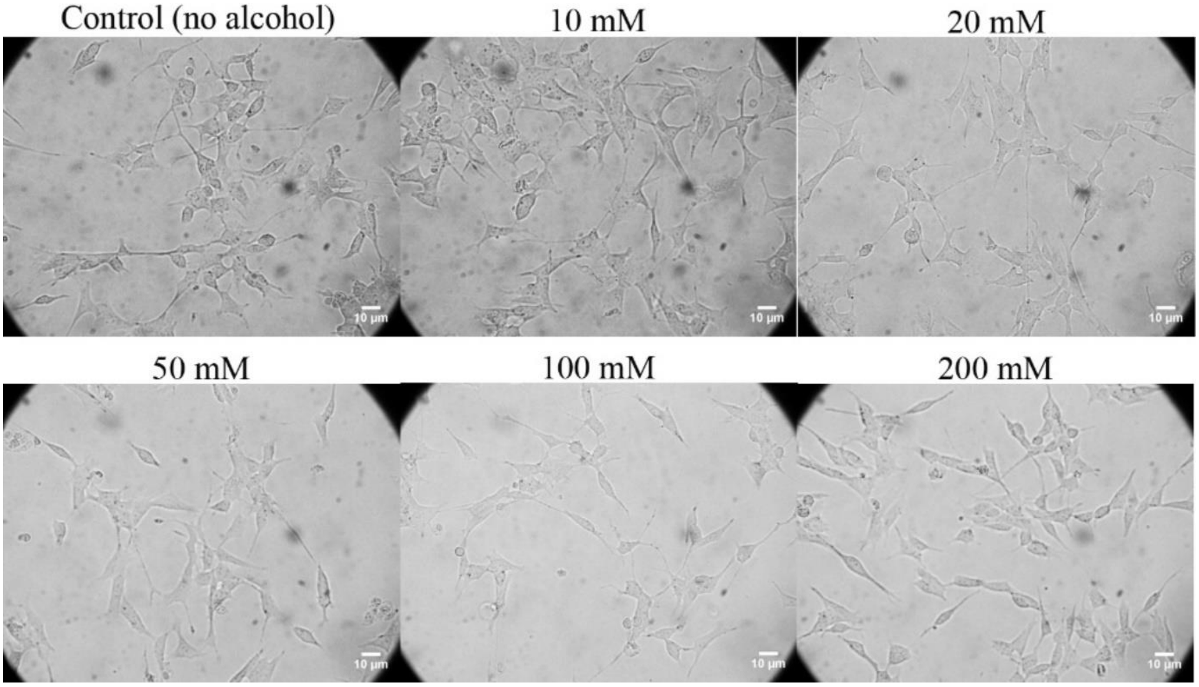

12 hour alcohol exposure

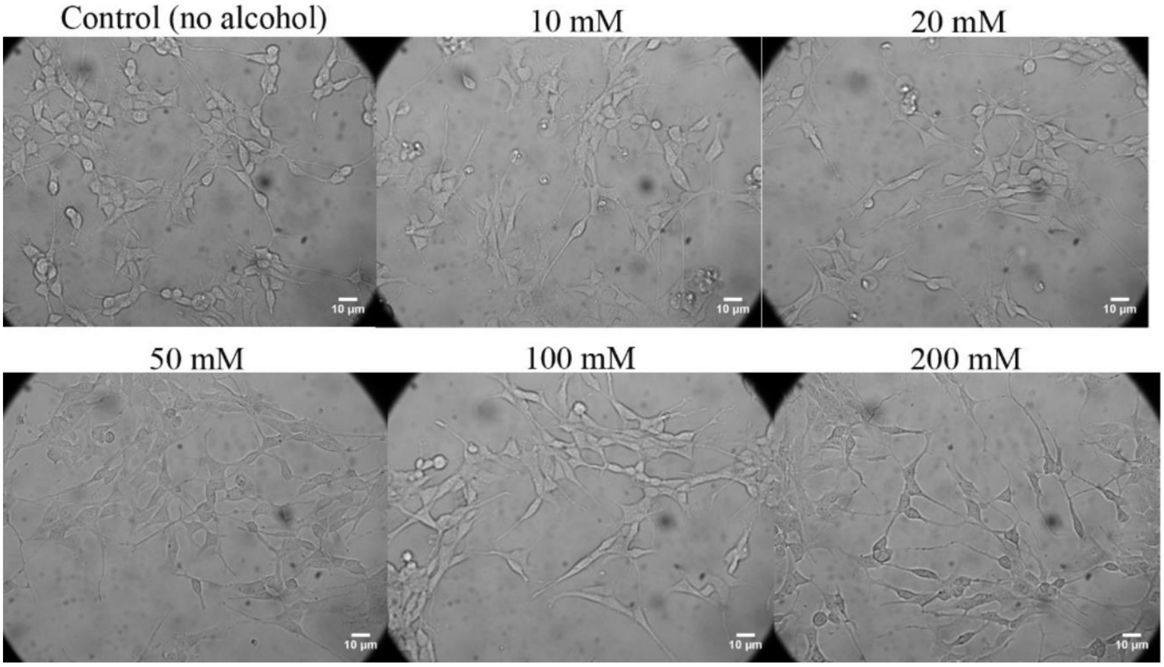

24 hour alcohol exposure

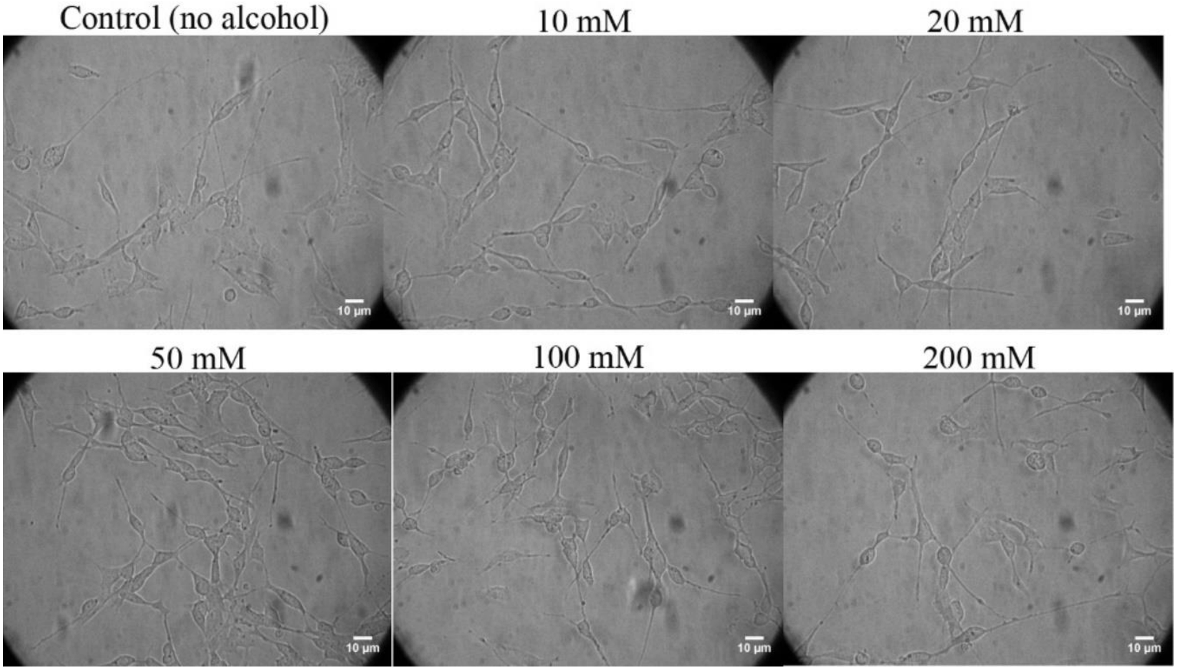

SH-SY5Y cells were differentiated in the presence of 0–200 mM alcohol for 3, 6, 12, and 24 hours, and images captured using a phase contrast microscope. Scale bar: 10 μm.

Supplementary Figure S2: Samples of original Western oxy-blots:

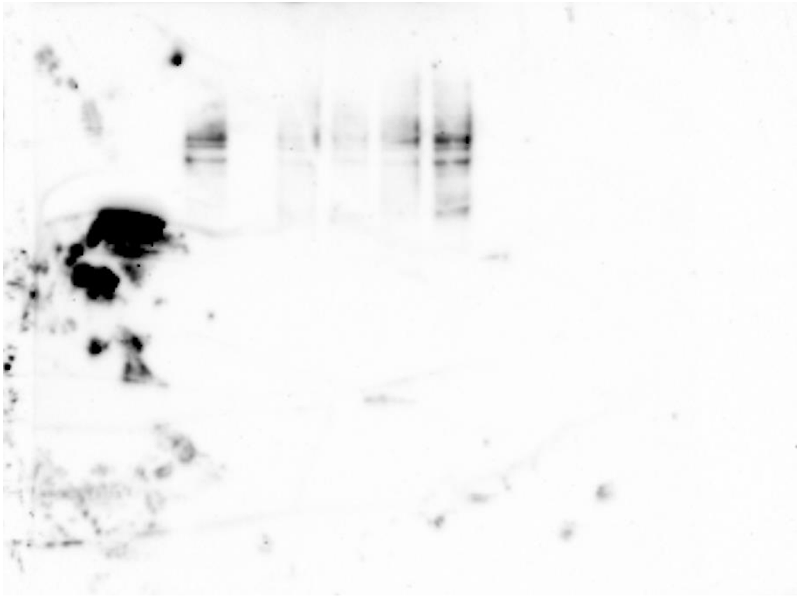

Full unedited blot for Figure 8 (left panel)

Lane 1, brain extract; Lane 2, 10 mM alcohol; Lane 3, 20 mM alcohol; Lane 4, 50 mM alcohol; Lane 5, 100 mM alcohol; Lane 6, 200 mM alcohol.

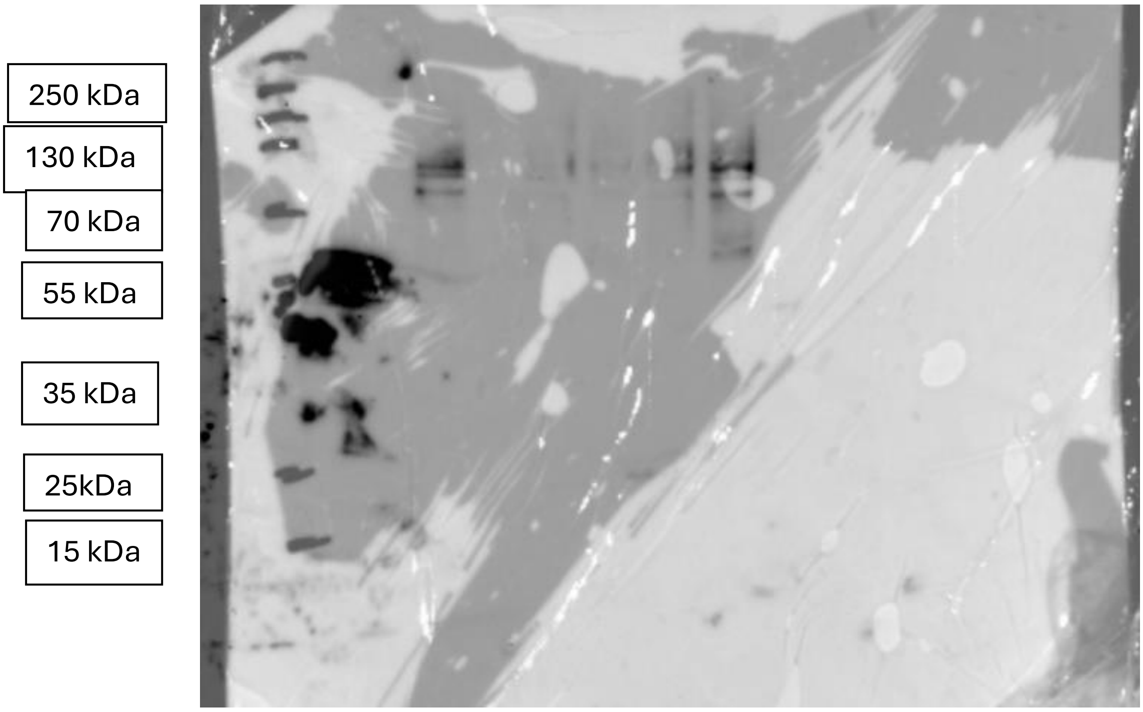

Full original unedited autoexposed blot used for Figure 8 (left panel) with blot co-exposed with markers for protein band sizing.

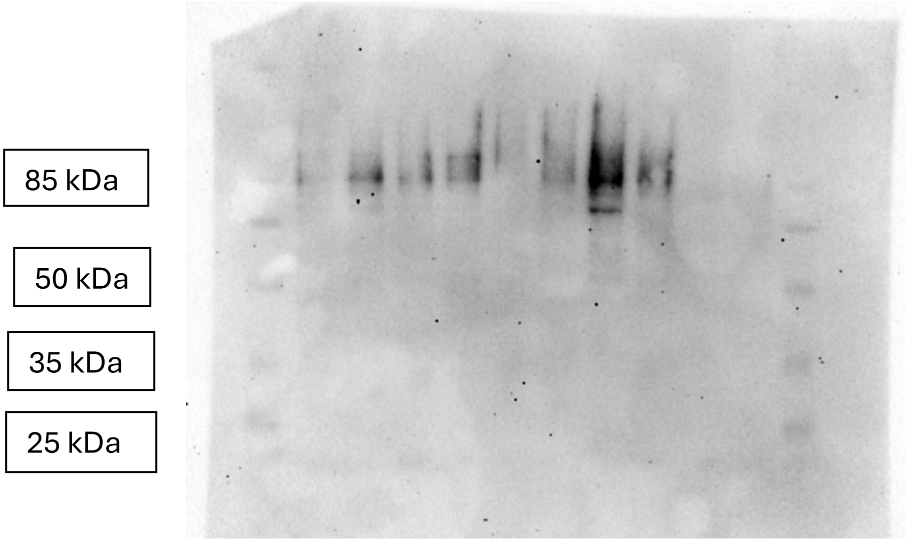

Full original unedited autoexposed blot used for Figure 8 (right panel). Lanes 1-5 used for the manuscript.

Lane 1, molecular weight markers; Lane 2, control brain 1; Lane 3, alcoholic brain 1; Lane 4, control brain 2; Lane 5, alcoholic brain 2; Lane 6, control brain 3; Lane 7, alcoholic brain 3; Lane 8, control brain 4; Lane 9, alcoholic brain 4; Lane 10, control brain 5; Lane 11, alcoholic brain 5; Lane 12, molecular weight markers.
